# Increased myositis and possible myocarditis in melanoma patients treated with immune checkpoint inhibitors in the COVID-19 era

**DOI:** 10.1007/s00262-024-03803-5

**Published:** 2024-10-05

**Authors:** Allison L. Gradone, Vincent T. Ma, Alexi Vasbinder, Leslie A. Fecher, Sarah Yentz, Salim S. Hayek, Christopher D. Lao

**Affiliations:** 1 Department of Internal Medicine, University of Michigan, Ann Arbor, MI USA; 2 Department of Medical Oncology, Sidney Kimmel Cancer Center, Thomas Jefferson University, Philadelphia, PA USA; 3 Division of Hematology and Oncology, Department of Internal Medicine, University of Michigan, Ann Arbor, MI USA; 4 Division of Hematology, Medical Oncology, and Palliative Care, Department of Internal Medicine, University of Wisconsin, Madison, WI USA; 5 Department of Dermatology, University of Wisconsin, Madison, WI USA; 6 Division of Cardiology, Department of Internal Medicine, University of Michigan, Ann Arbor, MI USA; 7 Division of Hematology and Oncology, Department of Internal Medicine, University of Michigan, Ann Arbor, MI USA; 8 Department of Dermatology, University of Michigan, Ann Arbor, MI USA

**Keywords:** Melanoma, Myocarditis, Immunotherapy, Myositis

## Abstract

**Background:**

Immune checkpoint inhibitor (ICI)-mediated myocarditis results in significant morbidity and mortality. At our institution, we noted an increased incidence of ICI-mediated myocarditis cases, leading to further investigation in our database of advanced melanoma patients treated with ICI therapy.

**Methods:**

A single-center, retrospective cohort analysis of patients with advanced melanoma identified cases of ICI-mediated myocarditis and myositis.

**Results:**

366 patients with advanced melanoma received a dose of ICI from September 2014 to October 2019. Of these patients, there were 0 cases of ICI-mediated myocarditis (0%, 95% CI 0%–1.0%) and 2 cases of ICI-mediated myositis (0.55%, 95% CI 0.07%–1.96%). From November 2019 to December 2021, an additional 246 patients with advanced melanoma were identified. Of these patients, 10 (4.1%, 95% CI 1.97%–7.35%) developed ICI-mediated myocarditis and 10 developed ICI-mediated myositis.

**Conclusion:**

Our study suggests an increase in prevalence of ICI-mediated muscle damage including myositis and myocarditis in the COVID-19 era. Differentiation of these patients and further risk stratification may allow for development of guidelines for nuanced management of this serious complication.

**Supplementary Information:**

The online version contains supplementary material available at 10.1007/s00262-024-03803-5.

## Introduction

Immune-related adverse events (irAEs) frequently occur in cancer patients treated with immune checkpoint inhibitors (ICI). In large trials of patients with advanced melanoma receiving ipilimumab, nivolumab/ipilimumab, nivolumab and pembrolizumab, ICI-mediated myocarditis was a rare complication with only 1 event out of 1186 patients (0.08%) enrolled [1, 2]. Other studies of patients receiving ICI have found rates ranging from 0.04 to 1.14% and up to a 50% mortality rate [3–8]. Since the start of the COVID-19 pandemic, we noted an increased incidence of ICI-mediated myocarditis cases at our institution, leading to further investigation in our database of advanced melanoma patients treated with ICI therapy.

## Methods

We identified 612 patients with histologically proven stage III or IV melanoma who were treated with ICI from September 2014 to December 2021 at the University of Michigan in Ann Arbor. Cases of ICI-mediated myocarditis and myositis were identified in our established IRB approved database and with manual search of electronic medical records with key terms utilizing DataDirect.

Cases of ICI-mediated myocarditis were identified based on clinical symptoms including shortness of breath and fatigue and associated laboratory abnormalities including elevated high-sensitivity troponin (HS-troponin), elevated aspartate aminotransferase (AST):alanine aminotransferase (ALT) ratio and elevated creatinine phosphokinase (CPK). Other diagnostic modalities including electrocardiogram, echocardiogram, and cardiac magnetic resonance were utilized to further characterize patients with suspected myocarditis consistent with American Society of Clinical Oncology (ASCO) 2021 Clinical Practice Guideline [9]. These cases were defined as definite, probable, or possible cases of myocarditis based on guidelines by Bonaca MP et al. [10].

To better understand the possible impact of increased diagnosis of ICI-myocarditis with the more recent use of high-sensitivity troponin, the rate of myositis was also investigated. ICI-mediated myositis cases were assessed by identifying patients who developed elevated creatine phosphokinase (CPK) and characteristic symptoms consistent with ASCO 2021 Clinical Practice Guideline [9].

The rates of myocarditis and myositis were estimated using the exact binomial 95% confidence intervals and compared using the Fisher’s exact test. The rates observed from November 2019 to December 2021 were compared to the previously published rate of 0.08% using the two-sided exact binomial test [1, 2].

**Fig. 1:**
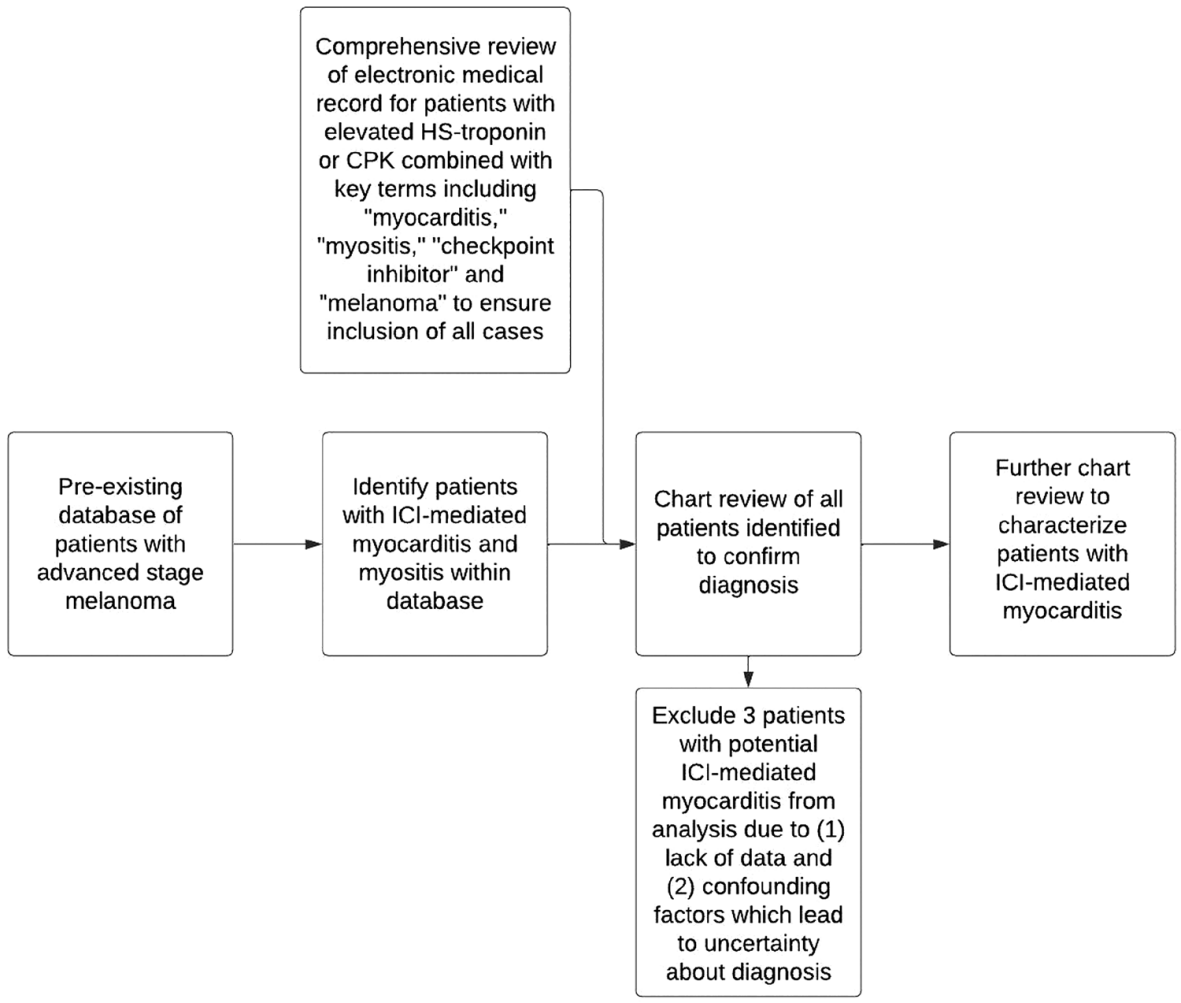
Methods flowchart

Three patients were excluded from analysis. One patient was treated for myocarditis at hospitals not associated with our institution limiting access to data. One patient was determined to have myocarditis attributed to avadomide. A third patient did develop elevated HS-troponin in setting of ICI-hepatitis; however, this was attributed to demand ischemia (Fig. 1).

## Results

**Fig. 2:**
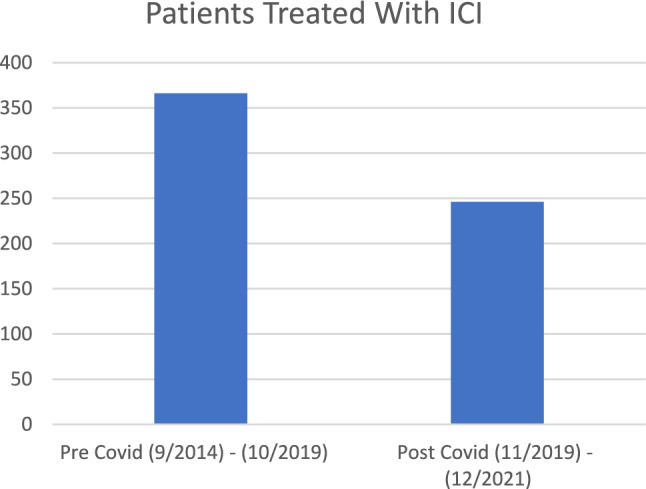
Patients treated with ICI

**Fig. 3:**
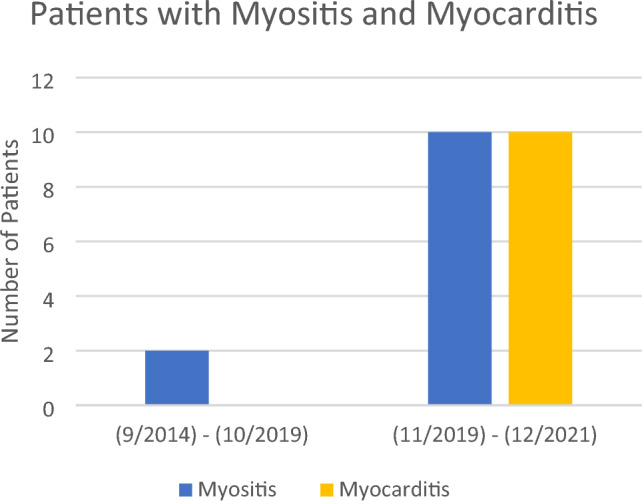
Patients with myositis and myocarditis

From September 2014 to December 2021, the rate of myocarditis was 1.6% (10/612) and the rate of myositis was 2.0% (12/612). Of the 366 patients with advanced melanoma that received their first dose of ICI between September 2014 and October 2019, there were 0 reported cases of ICI-mediated myocarditis (0%, 95% CI 0%–1.0%) and 2 cases of ICI-mediated myositis (0.55%, 95% CI 0.07%–1.96%). From November 2019 to December 2021, an additional 246 ICI-treated melanoma patients were identified. Of these patients, 10 developed ICI-mediated myocarditis and 10 (4.1%, 95% CI 1.97%–7.35%) developed ICI-mediated myositis (Table 1). We noted a statistical difference in the reported cases before and after November 2019 for ICI-mediated myocarditis (0% vs. 4.1%, *p* < 0.001) and ICI-mediated myositis (0.55% vs. 4.1%, *p* = 0.005). When compared to the rate of ICI-mediated myocarditis in patients with advanced melanoma treated with ipilimumab, nivolumab, nivolumab/ipilimumab, or pembrolizumab in previously published trials (Checkmate 067 and Keynote 006), the difference (0.08% vs. 4.1%) is also statistically significant (*p* = 0.019) [1, 2]. 1 patient was categorized as definite myocarditis, 1 patient was categorized as probable myocarditis, and 8 patients were categorized as possible myocarditis [10]. All patients were further classified as clinically significant but not fulminant [10]. Further clinical information is outlined in Supplementary Table 2 (Figs. 2, 3).

**Table 1:** Summary of results

	Total patients with ICI	Patients with diagnosis of myositis	Prevalence of myositis	Patients with diagnosis of myocarditis	Prevalence of myocarditis
Pre-COVID (9/1/2014–10/31/2019)	366	2	0.55% (95% CI 0.07–1.96%)	0	0% (95% CI 0%–1.0%)
Post-COVID (11/1/2019–12/2021)	246	10	4.1% (95% CI 1.97–7.35%)	10	4.1% (95% CI 1.97–7.35%)
Total	612	12	2.0%	10	1.6%

## Discussion

Since November 2019, around the time of early global detection of COVID-19, we noted an increased rate of ICI-myocarditis (4.1%). When comparing this cohort to the pre-COVID-19 cohort of patients (treated prior to November 2019) at our institution as well as historic data, there is a significant increase in the rate of patients who were diagnosed with ICI-mediated myocarditis [1, 2, 7, 8]. Previous studies have noted an increase in ICI-myocarditis prevalence, particularly from 2013 to 2018 [5, 6, 11]. This increase was attributed to increasing widespread use of ICI and higher clinical suspicion. However, our study notes an increase in prevalence dating from November 2019, the start of the COVID-19 era, which has not yet been reported.

The increased diagnosis rate of ICI-mediated myositis during the same time supports an overall increase in the prevalence of muscle injury. However, there is a possibility that some cases of myocarditis are misclassified and their presentations and outcomes may be more consistent with myositis rather than myocarditis, or that we are diagnosing subclinical or mild cases of myocarditis or other cardiac injury prior to fulminant presentation. Increased utilization of high sensitivity-troponin T assay (HS-troponin) as compared to troponin-I has been shown to be more sensitive in the identification of cardiac injury, which may lead to higher diagnostic accuracy and identification of more subtle presentations of myocarditis [12]. However, HS-troponin can be elevated in skeletal muscle disease, while troponin-I is rarely elevated in cases of myositis [12]. All cases in our analyses had elevations in both CPK and HS-troponin. Interestingly, all patients with at least 5 months of follow-up from myocarditis onset (*n* = 5) had persistently elevated HS-troponin despite normalization of CK, which has been described in ICI-mediated myocarditis [13]. How to confidently differentiate and risk stratify these clinical syndromes is not clear but important given the life-threatening nature of ICI-mediated myocarditis. This also has implications on risk stratifying patients to potentially re-challenge these patients with ICI.

Although more cases of ICI-mediated myocarditis will likely be reported due to higher clinical suspicion or awareness and increasing use of ICIs, our reported escalation in cases suggests an increase in prevalence of ICI-mediated muscle damage including myositis and myocarditis. As this series was not designed to establish a causal relationship between COVID-19 vaccination and infection and this datum was not able to be reliably obtained in a retrospective study, no conclusions can be drawn about the effect of the COVID-19 pandemic on this observation. However, both COVID-19 infections and vaccinations are associated with myocarditis [14–18]. Animal models have demonstrated that PD-1/PD-L1 inhibition can lead to exacerbation of viral myocarditis [19]. This area of inquiry would benefit from further investigation and may be elucidated as we learn more about COVID-19 and possible interactions with ICIs. Differentiation of these patients and further risk stratification may allow for development of guidelines for nuanced management of this serious complication.

## Limitations

Several limitations are recognized for this study. By design, this retrospective cohort analysis is unable to assess etiology, and data may be incomplete or subject to selection bias. Although, as noted above, all patients were diagnosed with ICI-myocarditis after ACS was ruled out as well as by expert consensus, the gold standard of cardiac biopsy was not done in any patient due to morbidity and mortality risks.

## Supplementary Information

Below is the link to the electronic supplementary material.

Supplementary file1 (DOCX 92 KB)

## Data Availability

No datasets were generated or analyzed during the current study.
