# Supplementary material for: Increased myositis and possible myocarditis in melanoma patients treated with immune checkpoint inhibitors in the COVID-19 era
Source: Cancer Immunol Immunother. 2024 Oct 5;73(12):259. doi: 10.1007/s00262-024-03803-5 (PMC11456101; doi:10.1007/s00262-024-03803-5)
Supplement: Supplementary file 1 — Supplementary file1 (DOCX 92 KB) [file 262_2024_3803_MOESM1_ESM.docx]

Table 2
